# A bibliometric analysis of college students' entrepreneurial intention from 2000 to 2020: Research trends and hotspots

**DOI:** 10.3389/fpsyg.2022.865629

**Published:** 2022-10-05

**Authors:** Gao Tingting, Yang Jiangfeng, Ye Yinghua

**Affiliations:** College of Education, Zhejiang University, Hangzhou, China

**Keywords:** college students, entrepreneurial intention, entrepreneurship education, research hotspots, research fronts

## Abstract

Based on 454 articles related to college students' entrepreneurial intention in the Web of Science Core Collection, this study explores the hotspots and trends of international research on college students' entrepreneurial intention by using a combination of coding and bibliometric analysis. The research hotspots are as follows: the theory of planned behavior is the main theoretical basis of these studies; entrepreneurship education is a more important predictor of college students' entrepreneurial intention, and this relationship is regulated by multiple variables; personal traits, several types of capital theories, social entrepreneurial intention, and quantitative research methods are also common. The research fronts include the following: systematic review of the field, continuous attention to the theory of planned behavior, and in-depth exploration of the differentiated influence of entrepreneurship education on entrepreneurial intention. Finally, we proposed research thinking and prospects related to research on undergraduates' entrepreneurial intention and entrepreneurship education.

## Introduction

With social development, more and more countries have realized the importance of entrepreneurship education (EE) and have paid attention to how to enhance entrepreneurial spirit through education (Fayolle and Gailly, 2015). Such education is considered to have an important impact on students' entrepreneurial development, which can improve entrepreneurial knowledge and skills and encourage engagement in entrepreneurial activities. It is used by governments as an effective strategy for promoting entrepreneurship, job creation, and poverty alleviation (Baluku et al., 2020). In the field of university entrepreneurship research, the entrepreneurial intention (EI) of university students, namely their degree of willingness to establish new businesses, has always been an important concern. On the one hand, the effect of EE can be measured by the change in students' EI. And on the other hand, according to the theory of planned behavior (TPB), behavioral intention is considered as an effective predictor of practical action, and people with high entrepreneurial intention are more likely to be potential entrepreneurs. To better understand the influencing factors of college students' EI, especially the influence of EE on EI, this study combined the coding and visual bibliometrics analytics to analyze the relevant studies on college students' entrepreneurial intentions in the past 20 years, and to identify the hotspots and fronts of the research on college students' EI and explore its implications for the effective cultivation of entrepreneurial talent in colleges and universities.

EI has a relatively long research history. Since the early 1980s, after Shapero's pioneering explorations of entrepreneurial events and the social dimensions of entrepreneurship, research on EI has grown rapidly. Thereafter, as social psychological theories were gradually integrated, such as Ajzen's theory of planned behavior and Bandura's self-efficacy theory, entrepreneurial intention research has been expanding and deepening in both theory and methods (Liñán and Fayolle, 2015).

## Data sources and research method

### Data sources

The Web of Science Core Collection was used for retrieving literature published from 2000 to 2020. Advanced searches with AK (keyword of the author) = (Entrepreneurial Intention), the type set to “article,” and the language restricted to “English,” were performed. As of October 31, 2020, a total of 685 records had been searched. After a careful reading of the abstracts of all the records, 470 records on students' EI were retained. In these records, 454 were related to college students (including MBA students), 16 to primary and middle school students, and finally, 454 records related to college students were reserved as the analysis records (454 records were used for preliminary coding and 450 records were finally imported into CiteSpace for analysis, for four records had not been exported). Thereafter, the bibliographic data were downloaded in the form of plain text.

**Figure 1:**
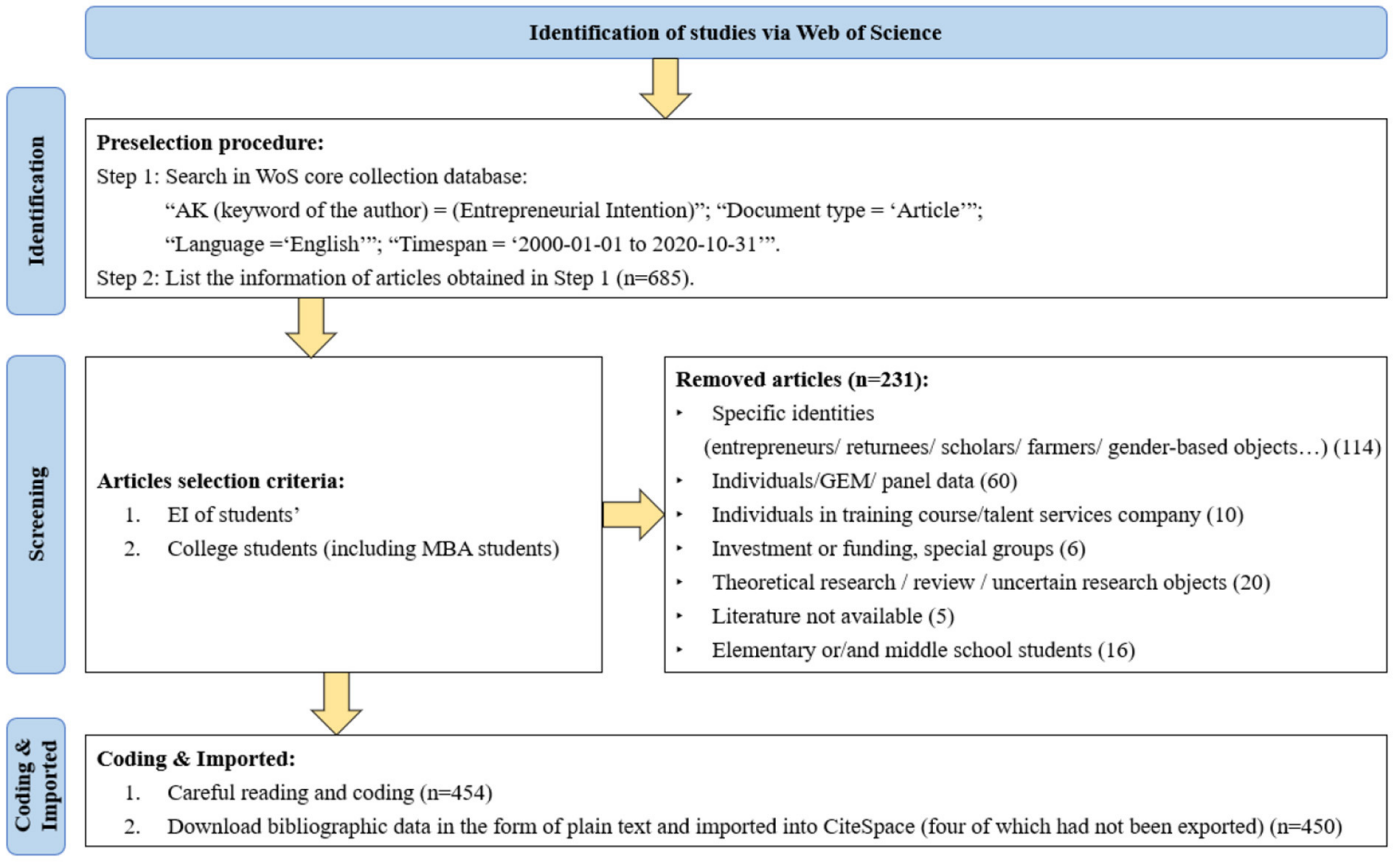
The flowchart of data selection.

To illustrate the process of identification, screening, and inclusion of literature, a flowchart diagram is provided in Figure 1. The identity and age of the subjects in each paper were clearly mentioned. After the initial screening, we also discussed uncertain data, and abandoned any imprecise papers.

### Research methods

This research mainly combined coding and CiteSpace, the latter is based on network analysis and visualization. For the coding part, we mainly analyzed the publication time, research sample, theoretical basis, research method, and sample size of the 454 papers. CiteSpace is developed by Dr. Chen and is based on the method of bibliometrics to visually analyze specific research fields and more intuitively identify the research fronts and hotspots of specific research fields (Chen, 2006). This research will explore the main research force (core authors, important journals), hotspots, and fronts in the field of college students' EI through the analysis of cooperation, co-occurrence, and co-citation of these papers and researchers.

## Research status

### Preliminary coding results of research on college students' EI

**Figure 2:**
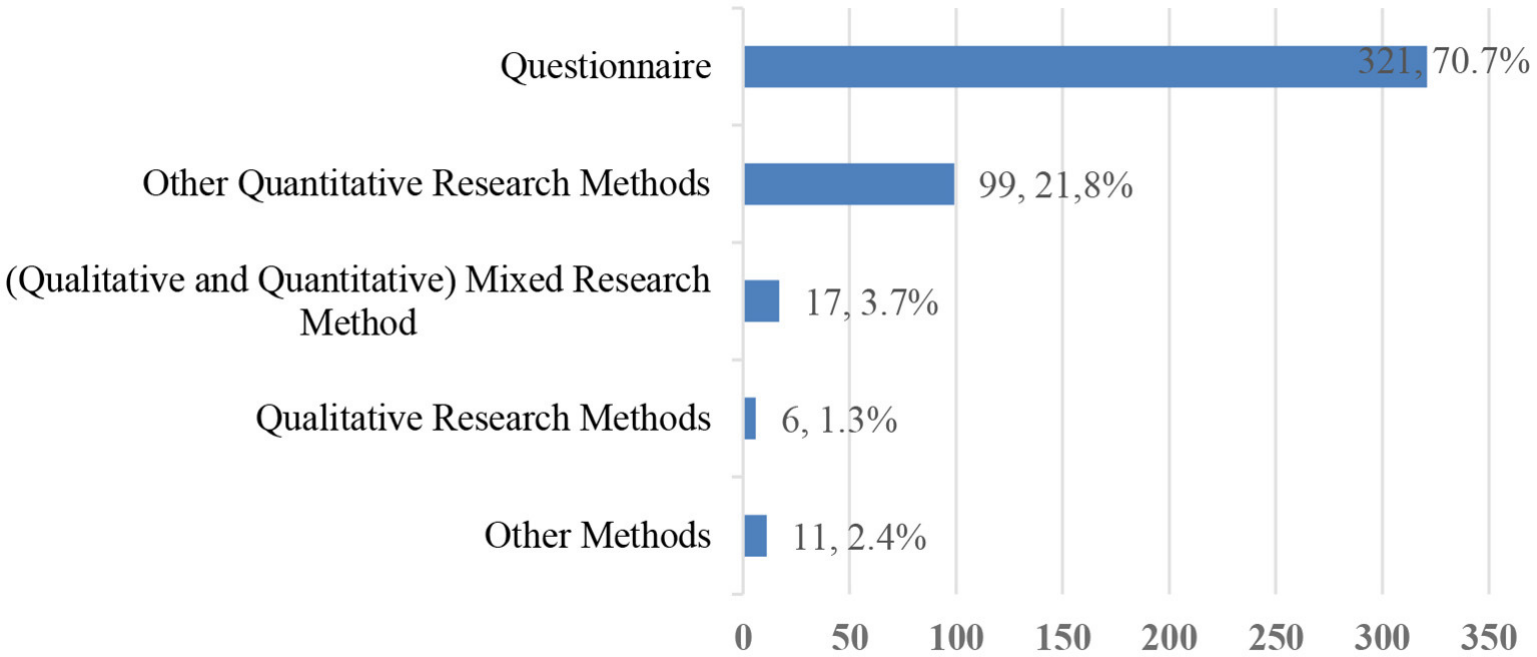
Method distribution of 454 articles.

This research first encoded the time, method, theoretical basis, and sample size of the research on college students' EI. In terms of the distribution of research time, the researcher searched the literature from January 1, 2000, to October 31, 2020, and found that the research on college students' EI mainly started in 2007. Before 2016, no more than 40 articles were published each year. The number of articles published in 2019 and 2020 increased sharply, with more than 100 articles published each year (Table 1). Quantitative research was the main research method, and <10% of the research used qualitative or mixed research methods (Figure 2). In terms of research theory, ~184 papers adopted TPB, accounting for around 41%. In addition, there were a small number of studies (<20 articles, respectively) based on the theory of personality traits, self-regulation and self-efficacy, social cognition, and entrepreneurial event models, etc. Some studies combined several theories to illustrate their point of view, while others did not involve an explicit theory. Of the research with a reported sample size, 89 articles had a sample size of 1–200, 191 had a sample size of 201–500, 93 had a sample size of 501–1,000, 39 had a sample size of 1,001–2,000, and 21 had a sample size of 2,000 or above. There were 21 articles that did not contain sample information.

**Table 1:** Time distribution of articles from 2000 to 2020.

**Year**	**2007**	**2008**	**2009**	**2010**	**2011**	**2012**	**2013**	**2014**	**2015**	**2016**	**2017**	**2018**	**2019**	**2020.10**	**Total**
**Count**	1	1	1	4	7	5	12	16	34	40	65	55	115	98	454

### Most prolific authors

The most prolific authors are important research forces in their fields, and their academic achievements promote the development of their fields. Through the analysis of the author network in CiteSpace, we explored the important authors in the field of college students' EI. The analysis results showed that authors such as Liñán (9), Liang (7), Garcia-Rodriguez (6), Farrukh (6), Shirokova (5), and Aloulou (5) had published five articles or more, and 12 authors, such as Otto, had published four articles.

### Important journals

In the co-citation analysis, the top 10 journals in the field of college students' EI research are listed in Table 2. “Entrepreneurship Theory and Practice” is the most cited journal in this field.

**Table 2:** Top 10 cited journals.

**Number**	**Count**	**Centrality**	**Cited journals**
1	424	0.38	Entrepreneurship Theory and Practice
2	414	0.19	Journal of Business Venturing
3	320	0.09	Organizational Behavior and Human Decision Processes
4	307	0.10	Journal of Small Business Management
5	260	0.02	Academy of Management Review
6	259	0.14	Entrepreneurship and Regional Development
7	252	0.11	Small Business Economics
8	249	0.04	International Entrepreneurship and Management Journal
9	248	0.12	Journal of Applied Psychology
10	239	0.04	Education and Training

### Document co-citation analysis

The knowledge base in research is composed of cited articles and will be relatively stable for a long period of time, highly cited literature represents more classic research results in related fields (Hou and Chen, 2007). In this research, with the help of document co-citation analysis, the knowledge base of the research field of undergraduates' EI was explored. Table 3 presents the basic information of the cited references.

**Table 3:** Basic information of document co-citation analysis.

	**Count**	**References**	**Title**
1	85	Liñán and Fayolle, 2015	A systematic literature review on entrepreneurial intentions: Citation, thematic analyses, and research agenda
2	83	Kautonen et al., 2015	Robustness of the Theory of Planned Behavior in predicting entrepreneurial intentions and actions
3	75	Schlaegel and Koenig, 2014	Determinants of entrepreneurial intent: A meta–analytic test and integration of competing models
4	71	Bae et al., 2014	The relationship between entrepreneurship education and entrepreneurial intentions: A meta-analytic review
5	58	Fayolle and Gailly, 2015	The impact of entrepreneurship education on entrepreneurial attitudes and intention: Hysteresis and persistence
6	56	Fayolle and Liñán, 2014	The future of research on entrepreneurial intentions
7	36	Rauch and Hulsink, 2015	Putting entrepreneurship education where the intention to act lies: an investigation into the impact of entrepreneurship education on entrepreneurial behavior
8	33	Karimi et al., 2016	The impact of entrepreneurship education: A study of Iranian students' entrepreneurial intentions and opportunity identification
9	33	Maresch et al., 2016	The impact of entrepreneurship education on the entrepreneurial intention of students in science and engineering vs. business studies university programs
10	33	Kautonen et al., 2013	Predicting entrepreneurial behavior: A test of the theory of planned behavior
11	33	Shirokova et al., 2016	Exploring the intention–behavior link in student entrepreneurship: Moderating effects of individual and environmental characteristics
12	31	Piperopoulos and Dimov, 2015	Burst bubbles or build steam? Entrepreneurship education, entrepreneurial self-efficacy, and entrepreneurial intentions
13	31	Zhang et al., 2014	The role of entrepreneurship education as a predictor of university students' entrepreneurial intention

### Highly cited references

A high frequency of citations indicates that the literature is representative of related fields. Table 4 presents the top 10 most cited articles on undergraduates' EI out of the 450 studies that were analyzed.

**Table 4:** Highly cited references on undergraduates' EI (Top 10).

	**Count**	**References**	**Title**
1	258	Liñán et al., 2011b	Regional variations in entrepreneurial cognitions: Start-up intentions of university students in Spain
2	235	Fitzsimmons and Douglas, 2011	Interaction between feasibility and desirability in the formation of entrepreneurial intentions
3	226	Kuckertz and Wagner, 2010	The influence of sustainability orientation on entrepreneurial intentions—Investigating the role of business experience
4	209	Liñán et al., 2011a	Factors affecting entrepreneurial intention levels: A role for education
5	140	Sánchez, 2011	University training for entrepreneurial competencies: Its impact on intention of venture creation
6	133	Laspita et al., 2012	Intergenerational transmission of entrepreneurial intentions
7	133	Díaz-García and Jiménez-Moreno, 2010	Entrepreneurial intention: the role of gender
8	124	Gupta et al., 2008	The effect of gender stereotype activation on entrepreneurial intentions
9	113	Zhang et al., 2014	The role of entrepreneurship education as a predictor of university students' entrepreneurial intention
10	113	Douglas, 2013	Reconstructing entrepreneurial intentions to identify predisposition for growth

### Keywords and clusters

Co-word analysis helps to explore research hotspots in the field of college students' EI by revealing the frequency of keywords (Zhao and Xu, 2010; Chen et al., 2015). Table 5 presents keywords that appeared more than 40 times.

**Table 5:** Keywords that appear more than 40 times.

**Number**	**Count**	**Centrality**	**Keywords**	**Number**	**Count**	**Centrality**	**Keywords**
1	356	0.34	Entrepreneurial intention	11	72	0.00	Entrepreneurship education
2	157	0.12	Self-efficacy	12	67	0.06	Business
3	148	0.01	Education	13	65	0.01	Entrepreneurship
4	133	0.00	Model	14	57	0.00	Intention
5	122	0.09	Impact	15	55	0.03	Perception
6	102	0.02	Student	16	53	0.00	Engineering student
7	86	0.03	Attitude	17	49	0.03	Motivation
8	86	0.06	Gender	18	47	0.02	Personality
9	76	0.01	Planned behavior	19	46	0.01	University student
10	73	0.08	Behavior	20	44	0.23	Determinant

Through analysis of the number of keywords, it was found that in addition to entrepreneurial intentions, self-efficacy (157), education (148), attitude (86), gender (86), entrepreneurship education (72), perception (55), motivation (49), personality (47), etc. are all important variables of concern in the study of college students' EI. Planned behavior (76) is also an important theoretical basis, and at the same time, research on the EI of engineering students (53) has attracted attention.

Clusters can help to further analyze hotspots in a research field. Cluster information is presented in Table 6, where the modularity is 0.7253 (Q > 0.3, indicating that a cluster's structure is significant), and the weighted mean silhouette is 0.9146 (S > 0.7, indicating that the cluster is convincing). The results showed that these clusters can better represent hotspots in the research field.

**Table 6:** Information of clusters.

**Cluster ID**	**Size**	**Silhouette**	**Lable (LLR)**
**#0**	33	0.99	Action-embedded pedagogy; New venture creation; Developing economy; Hong Kong; Comparative study
#1	32	0.86	Understanding entrepreneurship; South Korea; Planned behavior approach; Psychological trait; Family tradition
#2	29	0.92	Career intention; Career decision; Start-up intention; Regional variation; Entrepreneurial cognition
#3	27	0.92	Entrepreneurial passion; Personal characteristics; Entrepreneurial self-efficacy; Gender stereotype activation; Proactive personality
#4	24	0.76	Multi-group analysis; Indicator level; Gender difference; Entrepreneurial capital; Psychological capital
#5	23	0.92	Information systems view; Competence-social entrepreneurial intentions link; Subjective norm; Turkish case; Social entrepreneurial intention

## Research hotspots and fronts

### Research hotspots

Research hotspots generally refer to topics discussed in a group of internally related literature within a certain period. Generally, the results can be obtained based on indicators such as co-citation and co-word analysis (Pan and Wang, 2011). This research combined the relevant data obtained, including the preliminary coding results of the research, co-citation documents, cluster labels, and high-frequency keywords, to analyze and summarize the research hotspots of college students' EI from 2000 to 2020.

#### TPB is the main theoretical basis for this field of study

Among all the 454 articles about college students' EI, there are ~184 articles (about 41%) based on TPB, and eight of the 13 high co-citations also focus on this theory, indicating that it is an important knowledge foundation for research on EI among college students. At the same time, from the analysis of keywords, the frequency of factors related to TPB, such as self-efficacy, attitude, and planned behavior, are ranked relatively high. TPB is also the main theory presented in the representative literature of the first category of cluster labels. Therefore, it occupies an important position in the research on college students' EI and is the main theoretical basis for this research.

TPB believes that an individual's behavioral intention is predicted by three variables: attitude, subjective norms, and perceived behavioral control. Behavioral intention further predicts behavior and overall, the more positive the scores of these three variables, the stronger the individual's intention to implement a certain behavior, and the greater the possibility of the behavior (Kautonen et al., 2015). Kautonen et al. (2015) proved the relevance and robustness of TPB in predicting EI and subsequent start-up behavior based on longitudinal survey data from Austria and Finland in 2011 and 2012. This research addressed two weaknesses in the current research field of college students' EI: the limited sample scope and the scarcity of investigations and studies on the transformation of EI into actions. The results showed that attitude, subjective norms, and perceived behavior control can explain 59% of changes in entrepreneurial intention (usually between 30 and 45% in previous related studies). In addition, the results of a longitudinal study showed that the influence of entrepreneurial intention on entrepreneurial actions is robust, and the intention-behavior relationship remains unchanged regardless of a range of different demographic variables (Kautonen et al., 2015). The study by Kautonen et al. (2015) broke through the limitations of sample size and longitudinal investigation, fully proved the explanatory effect of TPB on EI and behavior, and is ranked second in the co-citation analysis. In addition to the overall predictive effects, the three predictors of PBL have different effects on EI. It has been suggested that attitude and perceived behavior control have more significant effects than subjective norms (Liñán et al., 2011a).

In addition to TPB, the entrepreneurial event model is also a more widely accepted and used model in EI research (Uysal and Güney, 2016). The two core elements of the model are perceived desirability (the attraction of being an entrepreneur) and perceived feasibility (the extent to which an individual thinks he/she can perform entrepreneurial actions), and they correspond to attitude and perceived behavioral control in PBL. Therefore, many studies have combined these two models. Apart from the individual effects of each variable, there are also representative studies that have explored the interactive effects of variables. Fitzsimmons and Douglas (2011) concluded that both perceived desirability and feasibility can positively predict EI. However, they found that the interactive effect of the two variables on EI is negative. In addition, perceived desirability has been proven to have a significant effect on EI, while perceived feasibility has no such effect (Zhang et al., 2014).

Although there are differences between different research results, overall, PBL is robust to the prediction of EI, and this predictive relationship is less affected by culture, so it is generally recognized in research on college students' EI and has become the main theoretical basis of the field.

#### EE is a more concerned predictor of college students' EI

EE refers to all educational processes or projects that may improve entrepreneurial skills or attitudes (Bae et al., 2014). There is a study that has subdivided EE into teaching on accounting, finance, marketing, management; teaching on competencies as personality traits and attitudes; business planning, and interaction with practice (Sánchez, 2011). Human capital theory and the theory of entrepreneurial self-efficacy provide support for the possible influence of EE on EI. Education is a core element of human capital theory. Factors such as the years of education received by an individual will have an impact on their subsequent career earnings. This effect also exists in the field of entrepreneurship. Among the 13 high co-citation documents, seven focused on the effect of EE on EI, indicating that EE is an important knowledge base in related research. Meanwhile, “education” as a high-frequency keyword, preceded only by “intention” and “self-efficacy,” was also the focus of the first two categories of clusters, indicating that EE is one of the hotspots in the research of college students' EI. The phrase, “entrepreneurship education has a small but significant impact on college students' EI, and this correlation is regulated by multiple types of variables,” can better summarize the relevant research results of this hotspot.

In a classic meta-analysis, researchers analyzed 73 studies with a total sample size of 37,285 and found that there was a significant but small correlation between EE and EI (Bae et al., 2014). More importantly, after controlling for pre-education EI, the correlation between EE and post-education EI was not significant (Bae et al., 2014). Some research results support the positive impact of EE on intentions. For example, based on PBL, some researchers combined experimental methods and questionnaires, and concluded that EE significantly affects start-up intentions and behaviors by improving attitude and perceived behavioral control, and EI mediates the effect of EE on subsequent behavior (Rauch and Hulsink, 2015). However, some studies found that the direct impact of EE on EI was not significant. This relationship can be regulated by different variables, such as the initial level of students' EI and prior exposure to entrepreneurship (Fayolle and Gailly, 2015).

#### The impact of EE on college students' EI is regulated by multiple variables

Many studies have focused on the variables moderating the relationship between EE and EI. The analysis carried out in these studies found that cluster labels or high-frequency keywords such as regional differences, personality characteristics, gender differences, and gender stereotypes as hotspots, are often studied as the moderating variables of EE affecting EI. By analyzing the representative literature, the moderating variables can be divided into different types, such as the individual-level factors, the characteristics or models of EE, cultural background, regional economic background, and the operation of EE. The research on their effects is shown in Table 7.

**Table 7:** Moderating variables between EE and EI.

**Entrepreneurship education—entrepreneurial intention**	**Sign of coefficient**	**Diagram**
■Moderator: Entrepreneurial experience		
Weak/inexistent or highly exposed	N.S.	/
■ Moderator: Initial entrepreneurial intention		
High	Negative	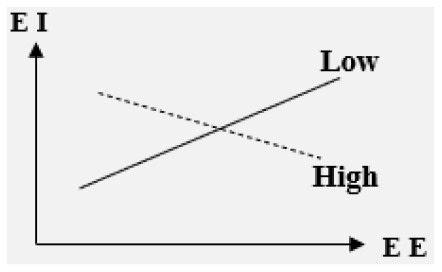
Low	Positive	
■ Moderator: Course type		
Elective course	Positive	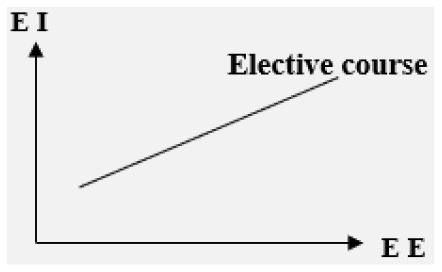
Compulsory course	N.S.	/
Practically oriented course (Entrepreneurial Self-Efficacy-EI)	Positive	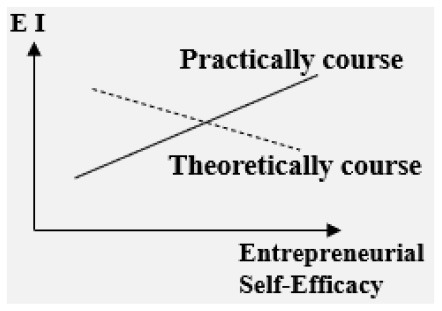
Theoretically oriented course (Entrepreneurial Self-Efficacy-EI)	Negative	
Semester format or workshop format	N.S.	/
■ Moderator: Course content		
Business planning or venture creation	N.S.	/
■ Moderator: Method of assessment		
Continuous assessment> Binary assessment	Positive	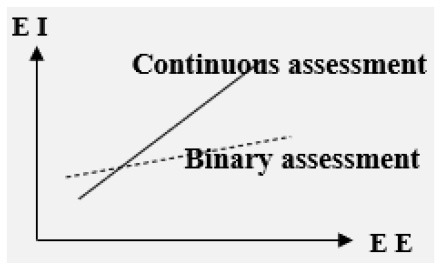
■ Moderator: Cultural context		
High in-group collectivistic> Low in-group collectivistic	Positive	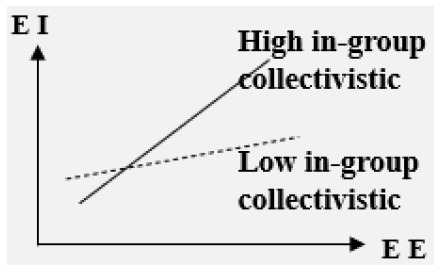
Low gender egalitarianism> High gender egalitarianism	Positive	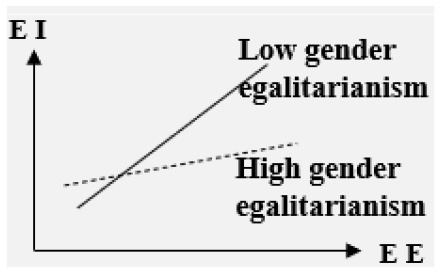
Low uncertainty avoidance> High uncertainty avoidance	Positive	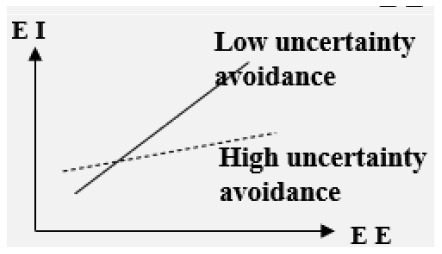

N.S., Not Significant.

The diagrams only show the direction of variables correlation.

**–Prior exposure to entrepreneurship**. Insufficient exposure to entrepreneurship may strengthen the influence of EE on students' perceived subjective norms and behavioral control, whereas highly exposed students may be negatively affected by EE (Fayolle and Gailly, 2015). But its effects on the EE–EI relationship may not be significant (Bae et al., 2014; Fayolle and Gailly, 2015).

**–The level of initial entrepreneurial intention**. There is a significant negative correlation, that is, the higher the level of initial entrepreneurial intention, the weaker the impact of EE on EI (EE can negatively affect students with initial entrepreneurial intention) (Fayolle and Gailly, 2015).

**–Entrepreneurship course type: elective course or compulsory course**. The influence of EE on EI is significant in elective courses, but is not significant in compulsory courses (Karimi et al., 2016).

**–Entrepreneurship course type: theoretically oriented course or practically oriented course**. The type of entrepreneurship course plays a moderating role in the relationship between entrepreneurial self-efficacy and EI. For theoretically oriented courses, the relationship is negative, and for practically oriented ones, the relationship is positive (Piperopoulos and Dimov, 2015).

**–Entrepreneurship course type: semester format or workshop format**. There is no significant impact on the relationship between EE and EI (Bae et al., 2014).

**–Entrepreneurship course content: business planning or venture creation**. There is no significant impact on the relationship between EE and EI (Bae et al., 2014).

**–Method of assessment**. EE has a stronger effect as a continuous assessment variable than as a binary variable (Bae et al., 2014).

**–Cultural context**. In the three cultural contexts of high in-group collectivism, low gender egalitarianism, and low uncertainty avoidance, EE has a stronger influence on EI (Bae et al., 2014).

Some variables have direct influences on the intensity of EI, including (As shown in Table 8):

**Table 8:** Direct influencing variables of EI.

**Direct influencing factors of entrepreneurial intention**	**Sign of coefficient**
■ Factor: Gender	
Men> Women (In general conditions)	Positive
■ Factor: Level of regional economic development	
Social valuation—EI (More developed regions)	Positive
Closer valuation—EI (Less developed regions)	Positive
■ Factor: Contextual motivations	
The openness-to-change values—EI (Malaysia and Indonesia)	Positive
The achievement values—EI (China)	Positive
■ Factor: Different types of entrepreneurial growth intentions	
Entrepreneurial self-efficacy—growth-oriented EI	Positive
Work enjoyment preference—growth-oriented EI	Negative
Risk tolerance—independence-oriented EI	Negative

**–Gender and gender stereotypes**. Researchers have proved the influence of gender on EI (Díaz-García and Jiménez-Moreno, 2010). Compared with women, men have stronger EI in general conditions (Gupta et al., 2008). And gender stereotypes also play a role in the process, when presented with gender-neutral information, there is no gender difference in the level of EI (Gupta et al., 2008).

**– Level of regional economic development**. In more developed regions, the social valuation of entrepreneurs is higher, and it has a positive impact on subjective norms and PBC. In less developed regions, the closer valuation is more important, as it can predict behavior attitudes and subjective norms (Liñán et al., 2011b).

**–Contextual motivations**. There are differences in the values held by college students in different contexts. For Malaysian and Indonesian university students, the openness-to-change values have greater influence on their EI. For Chinese university students, achievement values are more important (Looi, 2020).

**–Different types of entrepreneurial growth intentions**. Some researchers distinguish between growth-oriented and independence-oriented firms, and the former contribute more to societal benefits. The study results showed that entrepreneurial self-efficacy was significantly positively correlated with growth-oriented EI, and work enjoyment preference was significantly negatively correlated with growth-oriented entrepreneurial intention, risk tolerance and independence-oriented EI were negatively correlated (Douglas, 2013).

#### Other hotspots in the research field of college students' EI

According to the clusters, the research hotspots of college students' EI also include the following:

**The influence of personal traits on college students' EI has received continuous attention**. There are many representative citing documents in cluster #3 that explore the influence of college students' characteristics on EI. For example, Sun et al. (2020) used data from engineering students and concluded that creativity and risk-taking have a direct impact on EI, and the need for achievement and the locus of control indirectly affect EI. Li et al. (2020) used data from college students to explore the role of entrepreneurial alertness in entrepreneurial passion influencing EI and behavior. Syed et al. (2020) focused on the role of entrepreneurial passion, innovativeness, and curiosity in stimulating college students' EI. Entrepreneurship personality trait is an early concern in the field of research that affects EI. However, its impact is as uncertain as the impact of EE on EI. It is still a focus of attention in the field of factors of college students' EI.

**Several capital theories constitute the research foundation for college students' EI**. Capital theory has covered the development process of financial, human, social, and psychological capital. These types of capital are related to entrepreneurship start-up, team building, entrepreneurial performance, and the success or failure of entrepreneurship. Unger et al. (2011) used meta-analysis to analyze 70 studies (sample size = 2,4733), and the results showed that there is a significant but small correlation between human capital and entrepreneurial success. Some researchers (Mamun et al., 2016) took more than 400 female entrepreneurs in Malaysia as their research object and concluded that the social capital of female entrepreneurs can influence their entrepreneurial capabilities and ultimately affect entrepreneurial performance. They divided social capital into three dimensions: relational, structural, and cognitive (Mamun et al., 2016). The extension of the concept of psychological capital is richer and includes entrepreneurs' self-efficacy, optimism, hope and resilience, happiness, and social capabilities. Exploring the impact of EE on EI is in fact based on the exploration of human capital theory, and exploration based on the theory of self-efficacy can also be regarded as the exploration of psychological capital theory. These theories overlap to a certain extent in their connotations. In cluster #4, there is representative citing literature discussing capital theories in the study of college students' EI. Research by Turulja et al. (2020) showed that informal support from family and friends is positively correlated with EI, and can reduce the negative correlation between fear of failure and EI, while Zhao et al. (2020) focused on the impact of college students' psychological capital on EI. Using 1,914 Chinese college students as a research sample, it was shown that psychological capital has a significant indirect correlation with college students' EI only through traditional financial, human, and social capital.

**The social EI of college students has received more attention**. In the past 10 years, the importance of social entrepreneurship has gradually increased. Social entrepreneurship is a combination of social mission and entrepreneurial action, it is the solution or alleviation of social problems through the income derived from entrepreneurial operations in the market (Pärenson, 2011). Among the eight most representative citing articles in cluster #5, four were concerned with college students' social entrepreneurship. Igwe et al. (2020) used self-efficacy and subjective norms as moderators to explore the influence of networking competence on social entrepreneurial intention, and the results showed that the main effect of networking competence—social entrepreneurial intention is significant. Akhter et al. (2020) took 231 students from a public university in Bangladesh as the research sample and explored the influence of self-efficacy, social support, and educational support on college students' social entrepreneurial intention. There is also a study based on TPB and social cognitive career theory that explores the relationship between outcome expectations and social entrepreneurial intention (Luc, 2020). Liu et al. (2021) examined the influence of empathy, self-efficacy, perceived social support, moral obligation, and previous experience of social problems on social entrepreneurial intention. A total of 1,930 took part in the five survey studies, and the results showed that personality traits and social problem experience have an impact on intention through the mediation of entrepreneurial creativity and the above four factors (empathy, self-efficacy, perceived social support and moral obligation).

**Quantitative methods, especially questionnaire surveys, are the main research methods**. The coding method was adopted to make a preliminary and general classification of the main research methods of the 454 articles. Regarding the main research method that was adopted, questionnaires account for about 70%, <10% of the research used qualitative or mixed methods, and the questionnaire survey was the main method in undergraduates' EI research. The 10 highest citations in related research all used quantitative research methods. Six of them mainly used questionnaires, three used a combination of experiments and questionnaires, and one mainly used panel data. Although the quantitative method is the main research method for college students' EI, among the 13 co-citation documents focused on in this study, four are meta-analyses or literature reviews. This shows that the literature review provides a credible and relatively complete knowledge base for subsequent research.

### Research fronts

The research fronts in the related research field can be reflected by the documents cited in the recent publications of researchers (Chen, 2006). A “burst” clarifies research fronts and trends by examining the time distribution of the frequency of relevant content (Zhao and Xu, 2010). In this research, 11 documents with burst values greater than eight were retained, and we analyzed these burst articles to explore the research fronts of college students' EI. Table 9 shows the information of references with citation history and burst.

**Table 9:** The information of references with citation history and burst.

**Authors**	**Burst**	**Year**	**Years of burst**	**Title**	**Citation history**
Liñán et al.	8.06	2009	2011–2014	Development and Cross–Cultural Application of a Specific Instrument to Measure Entrepreneurial Intentions	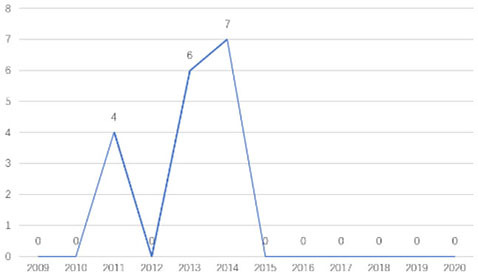
Liñán et al.	9.91	2011	2013–2016	Regional variations in entrepreneurial cognitions: Start-up intentions of university students in Spain	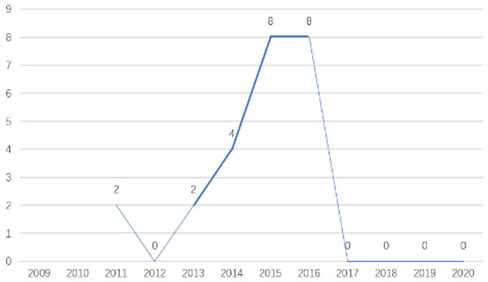
Shinnar et al.	8.22	2012	2014–2017	Entrepreneurial Perceptions and Intentions: The Role of Gender and Culture	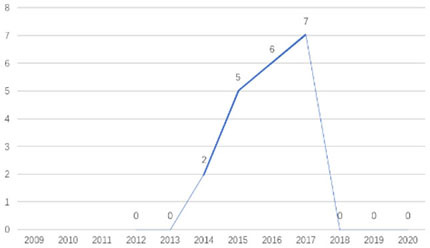
Fayolle et al.	8.96	2014	2015–2020	The future of research on entrepreneurial intentions	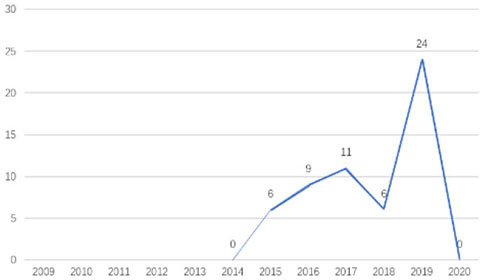
Kautonen et al.	12.18	2013	2015–2018	Predicting entrepreneurial behavior: A test of the theory of planned behavior	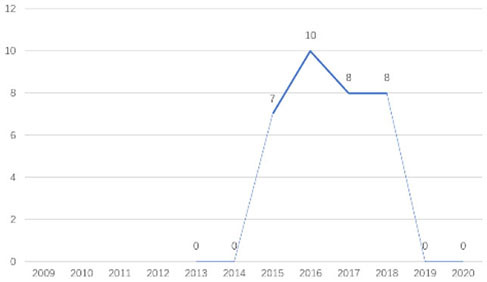
Schlaegel et al.	16.52	2014	2016–2020	Determinants of Entrepreneurial Intent: A Meta–Analytic Test and Integration of Competing Models	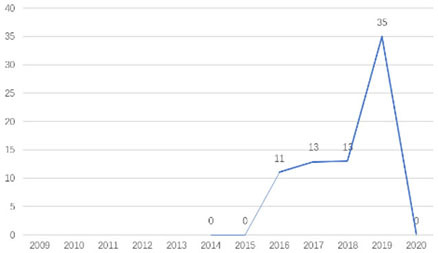
Bae et al.	15.29	2014	2016–2020	The Relationship between Entrepreneurship Education and Entrepreneurial Intentions: A Meta–Analytic Review	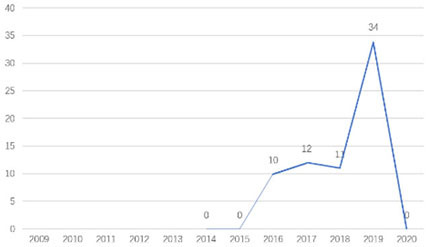
Zhang et al.	8.09	2014	2016–2018	The role of entrepreneurship education as a predictor of university students' entrepreneurial intention	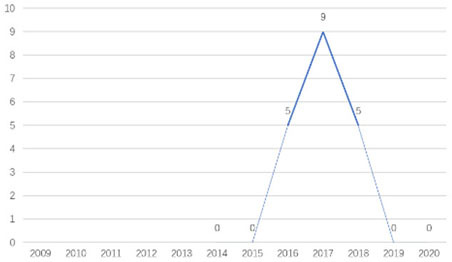
Liñán et al.	11.83	2015	2017–2020	A systematic literature review on entrepreneurial intentions: Citation, thematic analyses, and research agenda	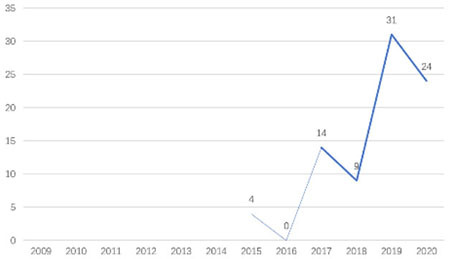
Kautonen et al.	8.71	2015	2018–2020	Robustness of the Theory of Planned Behavior in Predicting Entrepreneurial Intentions and Actions	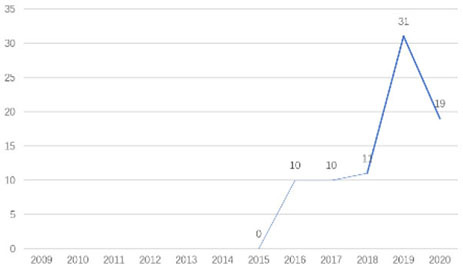
Maresch et al.	8.67	2016	2018–2020	The impact of entrepreneurship education on the entrepreneurial intention of students in science and engineering vs. business studies university programs	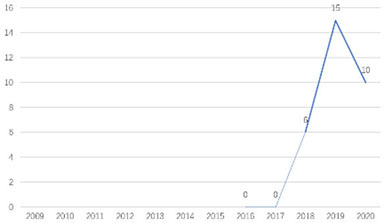

According to information from references with citation histories, the research fronts of college students' EI have experienced the following changes in research issues: exploration of EI measurement (2011–2014), attention to entrepreneurial cognitive factors (2013–2017), systematic review of EI research achievements and future trends (2015 to present), supporting analysis of TPB (2015 to present), and the impact of EE on intention (2016 to present). This research summarizes the fronts in the field based on several newly formed burst articles.

#### Systematic review of the achievements and problems in EI research, and analysis of future research trends

Among the recent burst articles, four are review or meta-analysis documents, indicating that it has always been the focus of this field to systematically review the research field and propose the prospects for future research. Fayolle and Liñán (2014) proposed some new perspectives for the research of EI: (a) focus on the core EI model and clarify the theoretical and methodological issues, (b) focus on the personal-level factors in predicting EI, (c) explore the relationship between EE and EI, (d) focus on the role of context and institution in EI, and (e) explore the connection between the entrepreneurial process and the intention–behavior link.

#### Continuous attention on TPB

Research based on TPB has been conducted for a long time. According to the burst information, this field will continue to pay attention to this theory.

#### Exploration of the different impacts of EE on EI

The impact of EE on EI is not only a hotspot but also a front. From the perspective of burst information, in addition to focusing on the overall impact of EE on intention, more attention has recently been paid to the differentiated performance of this impact relationship, such as the differences in the impact on students of different majors.

## Research thinking

Based on 454 articles on college students' EI, this study systematically reviewed the research status, hotspots and fronts in this field, and proposes corresponding thinking and prospects as set out below.

### Solidification and generalization of theory

In the extant studies on college students' EI, about 40% use TPB as the theoretical basis. Although there are other theories, such as entrepreneurial events, self-efficacy, and personality models, the theoretical basis is, in general, relatively solidified and unitary. Theoretical solidification implies several meanings:

The first is the generalization of the concept of EI. Ajzen's theory of planned behavior can generally explain intention and behavior, but there are differences between entrepreneurial behavior and general behavior. One of these differences lies in its high innovation, which determines the complexity of its influencing process. In addition to the three factors of attitude, subjective norms, and perceived behavioral control, the interaction of other factors, such as context, seems to be included less in the model.

The second is the solidification of entrepreneurial type. There may have differences in the main predictors of different entrepreneurship types (such as business entrepreneurship, social entrepreneurship, growth-oriented, and independence-oriented entrepreneurship), which have not been reflected well in previous studies.

These factors also make it rare for new theories to be developed in research on college students' EI.

Since individuals with the same behavioral intention may not perform equally, some studies added new variables to their theoretical model to enhance explanation (Duan and Jiang, 2008). For example, Baluku et al. (2020) supplemented TPB from the perspective of positive psychology, focusing on the impact of positive psychological attributes (proactive personality and psychological capital) on college students' EI; Ip et al. (2021) used the revised Hockerts' model based on TPB, etc. Other researchers explored self-employment using other theories, such as Barba-Sánchez and Atienza-Sahuquillo (2017), believed that expectation theory provided an excellent framework for understanding why and how people choose to become entrepreneurs, and they emphasized the importance of EI. In addition, TPB can not only explain and predict behavior, it can be used for behavioral interventions, achieving the goals of changing behavior by influencing attitudes, subjective norms, and perceived behavior control. However, many studies have focused only on its predictive effects (Duan and Jiang, 2008).

### Method simplification

The simplification of methods is mainly manifested in the simplification of research techniques, samples, and data forms.

First, the preliminary coding results showed that the questionnaire survey method is the main method used in this field. It has the advantage of collecting large sample data in a short time and obtaining relevant research conclusions. However, one of its disadvantages is that the investigation is not in-depth, and it is difficult to understand internal deep-seated mechanisms, and some differentiated exploration may be ignored.

Second, the simplification of methods is also reflected in the research samples. Many studies took college students as research objects but did not consider the differences in their professional composition, knowledge base, region, economic level, school, culture, and other factors. In fact, differences within the students are sufficient to affect the impact of EE on intention. For example, there may be great differences between non-quasi-entrepreneurial and quasi-entrepreneurial college students. In addition, as the number of “returnee” college graduates is increasing year by year, more attention should be paid to the EI of “new generation returnees.” At present, there is a limited amount of literature on EI in this group.

Finally, in terms of data form, long-term longitudinal and experimental data are relatively scarce.

### Superficial understanding and measurement of EE

EE in colleges and universities first appeared in the United States and the United Kingdom in the 1940s, and initially, it was mainly found in business schools (Hoppe et al., 2017). It has experienced conceptual development from entrepreneurship education to entrepreneurial learning; in terms of teaching content, knowledge, skills, attitude, and spirit have all received varying degrees of attention; regarding teaching methods, both theory and practice are emphasized, and teaching goals are gradually becoming diversified (Hytti and O'Gorman, 2004; Hoppe et al., 2017).

The goals of EE can be divided into three categories (Hytti and O'Gorman, 2004): First, it forms a broad understanding of entrepreneurship, especially the role of entrepreneurship and entrepreneurs in modern economies and societies. It can be carried out at different levels of education, such as primary school, secondary school, and university. Second, “learning to become entrepreneurial,” and to deal with individuals' needs to be responsible for their own studies, careers, and lives, which can be realized by providing information through education and training interventions. Third, learning how to become an entrepreneur by learning how to start a business. Students can learn the basic skills and information needed to become entrepreneurs by setting up small firms in a classroom environment.

According to the initial classification of Jamieson, Hoppe et al. (2017) summarized the types of EE into four categories: For/In/Through/About (FITA), including education in different modules such as entrepreneurship skills, practice, and knowledge foundation. It should be said that whether it is the concept of EE itself, or the content, goals, and methods of education are diversified. However, in past research and practice, the understanding and measurement of EE have been relatively superficial. Many studies only use it as a “binary variable” to assess, far from gaining a deep understanding and assessment of EE. This may be one of the main reasons for the contradictory results regarding the impact of EE on EI in different studies.

## Research conclusion

By exploring the status and problems of research on college students' EI, this study makes suggestions about theoretical development and measurement optimization, which, although not analyzed in depth, are conducive to improving stakeholders' attention and innovation regarding relevant theories and methods. Conclusions on whether EE is positive related to entrepreneurial behavior are inconsistent; therefore, it is more appropriate to use “small but significant” to describe the impact of EE on EI. Although relevant research has used a combination of TPB and other related models to study EI, there is still a gap between these theoretical models and current entrepreneurial realities (Barba-Sánchez and Atienza-Sahuquillo, 2018). Future research should pay greater attention to the intention model's predictive validity; TPB's intervention role also requires more analysis.

In addition to EE, a large part of this study emphasizes the role of moderating variables and other variables affecting EI. The differences in EE's explanation of EI may also be attributed to these variables, which make the measurement of EE's effectiveness differ from actual ones. Future research can explore additional potential moderators between EE and EI. Based on the complexity and richness of influencing factors, attention should be paid to controlling related variables in the process of exploring effectiveness; the implementation of EE needs to be more targeted, and measures should be taken according to the characteristics of students. In addition, future research trends are clearly presented through the bibliometric analysis, helping researchers to rapidly grasp the knowledge base and research hotspots, which contributes to EI research.

## Data availability statement

The datasets presented in this study can be found in online repositories. The names of the repository/repositories and accession number(s) can be found in the article/supplementary material.

## Author contributions

All the work of the paper is contributed together by YY, GT, and YJ. More research design and analysis were done by YY. More work on research methods by GT. More translation work by YJ and GT. All authors contributed to the article and approved the submitted version.

## Funding

The funding was provided by the National Educational Science Planning Project the Research of Influential Mechanism and Policy on New Generation of Returnees' Entrepreneurial Intention and Action (Grant No. BIA170195).

## Conflict of interest

The authors declare that the research was conducted in the absence of any commercial or financial relationships that could be construed as a potential conflict of interest.

## Publisher's note

All claims expressed in this article are solely those of the authors and do not necessarily represent those of their affiliated organizations, or those of the publisher, the editors and the reviewers. Any product that may be evaluated in this article, or claim that may be made by its manufacturer, is not guaranteed or endorsed by the publisher.
